# Antibacterial and antivirulence factor activities of protein hydrolysates from Phatthalung Sangyod rice (*Oryza sativa* L.) seeds against zoonotic and foodborne pathogens

**DOI:** 10.14202/vetworld.2023.2002-2015

**Published:** 2023-10-07

**Authors:** Prawit Rodjan, Suthinee Sangkanu, Watcharapong Mitsuwan, Monsicha Pongpom, Phirabhat Saengsawang, Irma Tedja, Jarunet Lamai, Kritsada Pruksaphon, Juthatip Jeenkeawpieam

**Affiliations:** 1 School of Agricultural Technology and Food Industry, Walailak University, Nakhon Si Thammarat, 80160, Thailand; 2 Center of Excellence in Innovation of Essential Oil and Bio-active Compound, Walailak University, Nakhon Si Thammarat, 80160, Thailand; 3 Department of Pharmacognosy and Pharmaceutical Botany, Faculty of Pharmaceutical Sciences, Prince of Songkla University, Hat Yai 90112, Songkhla, Thailand; 4 Akkhraratchakumari Veterinary College, Walailak University, Nakhon Si Thammarat, 80160, Thailand; 5 One Health Research Center, Walailak University, Nakhon Si Thammarat, 80160, Thailand; 6 Department of Microbiology, Faculty of Medicine, Chiang Mai University, Chiang Mai 50200, Thailand; 7 Department of Biochemistry and Molecular Biology, Monash Biomedicine Discovery Institute, Monash University, Victoria 3800, Australia; 8 Department of Medical Technology, School of Allied Health Sciences, Walailak University, Nakhon Si Thammarat, 80160, Thailand

**Keywords:** antibacterial peptide, foodborne pathogens, Phatthalung Sangyod rice, protein hydrolysate, zoonotic

## Abstract

**Background and Aim:**

Antimicrobial resistance is an emerging public health threat. Foodborne illnesses are typically caused by bacteria, such as *Escherichia coli*, *Pseudomonas aeruginosa*, *Bacillus cereus*, and *Staphylococcus aureus*, which are frequently resistant to common antimicrobial agents. Rice is a staple grain in most parts of the world. Our previous work showed that Phatthalung Sangyod rice seed protein hydrolysates (SYPs), especially SYP4, exhibit antifungal activity against several fungal species that are pathogenic for both humans and animals and are non-cytotoxic to animal red blood cells. In this study, we aimed to determine the effects of the bioactive peptides in SYPs against several pathogenic bacteria in humans and animals.

**Materials and Methods:**

After isolating SYP1, it was treated as follows: heated (SYP2), and hydrolyzed using pepsin (SYP3), and proteinase K (SYP4). Then, we used 500 μg of protein to evaluate the antibacterial effects on four pathogenic bacteria, including *E. coli*, *P. aeruginosa*, *B. cereus*, and *S. aureus*, using agar well diffusion. Using a broth microdilution assay, we determined the minimum inhibitory and bactericidal concentration (MIC and MBC, respectively) values of active SYPs. Using the agar well diffusion and microtube incubation methods, we also assessed the inhibitory effects of SYPs on the bacterial quorum sensing (QS) activity of *Chromobacterium violaceum*. Sangyod rice seed protein hydrolysates were evaluated for their ability to inhibit the biofilm formation of bacterial cells by a crytal violet assay. Furthermore, using the dropping method, we tested the inhibitory effects of SYPs on the bacterial pigments pyocyanin in *P. aeruginosa* and staphyloxanthin in *S. aureus*.

**Results:**

Our results showed that the crude protein lysate (SYP1) did not exhibit antibacterial activity against any of the test bacteria. Intriguingly, after boiling (SYP2) and enzymatic hydrolysis (SYP3 and SYP4), the protein hydrolysates were transformed into bioactive peptides and displayed antibacterial properties against all of the test bacteria at a concentration of 500 μg as determined by agar well diffusion. SYP4 demonstrated the highest antibacterial activity as it completely inhibited all test strains, with inhibition zones ranging from 16.88 ± 0.25 to 21.25 ± 0.5 mm, and also yielded the highest MIC/MBC values against *P. aeruginosa*, *B. cereus*, and *E. coli*, at 256 and >256 μg/mL, respectively. We observed that at least 256 μg/mL of SYP4 is required to exhibit optimal antibacterial activity. At 16–128 μg/mL, it exhibited antibiofilm activity against *S. aureus*. Furthermore, at 256 μg/mL, SYP4 inhibited pyocyanin in *P. aeruginosa* and staphyloxanthin in *S. aureus*. Although SYP2 and SYP3 displayed weak antibacterial activity and their MIC values could not be obtained for all bacteria, they showed strong QS inhibition in *C. violaceum* at 256 μg protein. Moreover, SYP2 and SYP3, at a minimum concentration of 32 μg/mL, significantly reduced violacein production. SYP3 also showed biofilm reduction activity on *S. aureus* at least 16–512 μg/mL.

**Conclusion:**

Sangyod Phatthalung protein hydrolysates exerted excellent inhibitory effects against the growth of bacteria and their virulence factors, such as QS, biofilm formation, and/or pigment production. These factors include zoonotic and foodborne pathogens. Therefore, daily consumption of Sangyod Phatthalung rice might reduce the risk of bacterial pathogenesis and foodborne diseases. In conclusion, functional foods or alternate methods of treating bacterial illnesses may be developed in humans and animals.

## Introduction

The Centers for Disease Control and Prevention estimated that approximately 48 million people worldwide are affected by foodborne illnesses. Of these, 128,000 are hospitalized, and 3000 die [1]. Therefore, ensuring food safety is crucial to prevent food spoilage and food poisoning caused by various foodborne zoonotic pathogens, including bacteria, viruses, fungi, and parasites [1–3]. *Staphylococcus aureus*, *Salmonella* species, *Campylobacter* species, *Listeria monocytogenes*, and *Escherichia coli* are among the major bacterial foodborne pathogens [1–5]. *Pseudomonas aeruginosa* commonly also causes food infections as it is highly adaptable and can grow at low temperatures [6]. These bacteria might develop antibiotic resistance over time, posing significant threats to public health and the economy [1, 7, 8]. Therefore, researchers are actively searching for novel antibiotics, particularly from natural sources like edible grains, to combat resistant pathogens [9], as synthetic compounds can be potentially hazardous [9]. As a result, there is a growing interest in natural antimicrobials derived from food.

One such natural source is Sangyod rice (*Oryza sativa* L.), a Thai Geographical Indication native to Phatthalung, Thailand [10]. It is rich in nutrients, including iron, calcium, phosphorus, proteins, carbohydrates, and vitamins B1, B2, and B6 [10]. Recent studies have explored the phytochemical properties of Sangyod rice seed and bran extracts, demonstrating their antibacterial effects against certain pathogenic bacteria [11, 12]. Studies have also shown that the Sangyod rice seed proteins can be a potential cost-effective protein substitute for humans and animals [13, 14]. In the gastrointestinal system, these proteins are broken down into beneficial bioactive peptides [15, 16]. However, due to their weak water solubility, their use in the food industry is limited. Protein hydrolysis is a sustainable method to enhance their functional qualities [17–20]. Despite previous studies on rice protein hydrolysates, little information is available about Sangyod rice seed protein hydrolysates, as only pepsin-hydrolyzed proteins have been reported. Although it can inhibit the growth of human pathogenic bacteria, such as *S. aureus*, *P. aeruginosa*, and *E. coli*, its mechanism of action and antivirulence effect on these bacteria is unclear [21]. In addition, our earlier findings suggested that the proteinase K-hydrolyzed Sangyod rice seed extract, SYP4, in particular, possesses bioactive peptides against pathogenic fungi. Further, it showed no hemolytic activity against canine erythrocytes [22].

This study aimed to determine the effects of the bioactive peptides from the Phatthalung Sangyod Rice (*O. sativa* L.) seed protein hydrolysates (SYP) on zoonotic and foodborne pathogens, including *S. aureus*, *B. cereus*, *P. aeruginosa*, and *E. coli*. Based on our findings, SYPs might have antibacterial and antivirulence properties against these pathogens.

## Materials and Methods

### Ethical approval

This study does not require ethical approval because the study was conducted *in vitro*.

### Study period and location

The study was conducted from June 2022 to January 2023 at Walailak University in Nakorn Si Thammarat, Thailand. The rice field was in the province of Phatthalung. Its latitude and longitude are 7° 36’ 33.59” N and 100° 04’ 13.20” E, respectively, and it is situated in a tropical climate zone. Protein preparation and extraction, antibacterial activity testing, and antibacterial virulence factor activity of the extracts were studied at Walailak University in Nakorn Si Thammarat, Thailand.

### Plant materials

Samples of Sangyod rice seeds were purchased from an organic Sangyod rice field in the Phattalung province, Thailand.

### Bacterial strains and growth conditions

*Bacillus cereus* WU22001 was provided by the microbiology laboratory at the Center for Scientific and Technology Equipment, Walailak University, Thailand. We also used *P. aeruginosa* ATCC27853, *E. coli* ATCC25922, and *S. aureus* ATCC25923). These were purchased from the American Type Culture Collection (Thermo Scientific, USA). Tryptic soy broth (TSB) (Difco, Claix, France) was used to cultivate a single colony of each bacterium in tryptic soy agar (TSA) (Difco). Bacterial samples were stored at −80°C in TSB with 20% glycerol until use.

### Preparation and isolation of SYPs

First, 50 g of Sangyod rice seed was ground into a fine powder, homogenized in 10 mM sodium acetate buffer (CH_3_COONa) (pH 5.2) with 0.5% phenolic-polyphenol pyrrolidine (PVP), and centrifuged at 4,500× *g* at 4°C for 20 min to remove the PVP compound-complex [22–24]. The collected supernatant was known as SYP1 and its protein concentration was determined. SYP1 was boiled at 100°C for 10 min, pepsin-hydrolyzed at a pH of 3.7, and hydrolyzed with proteinase K in 1× phosphate-buffered saline (PBS) (pH 7.4) to give SYP2, SYP3, and SYP4, respectively [22–24].

### Determination of protein concentration

The protein concentration was determined using the Bradford assay with bovine serum albumin (BSA) as a standard in a 96-well microplate [25]. This assay is based on the binding of the proteins to Coomassie Brilliant Blue G-250 dye, resulting in a shift in absorbance from 465 to 595 nm. The experiments were conducted as previously described by Jeenkeawpieam *et al*. [22–24] with slight modifications. A test well was filled with 5 μL of rice protein (triplicate). Bovine serum albumin was diluted to the final amounts of 0, 2, 4, 6, 8, and 10 μg in 5 μL for the calibration curve, and 5 μL of distilled water was used as blank. After adding 200 μL of the Bradford protein reagent (Bio-Rad Protein Assay; dye reagent concentrate, Bio-Rad, Hercules, CA, USA) into each well, the mixture was incubated at 25°C for 10 min and the absorbance was measured at 595 nm using a microplate reader (BioTek™ Synergy™ H4 Hybrid Microplate Reader, Winooski, VT, USA). A standard graph was generated using the BSA standard’s absolute absorbance (optical density [OD]_BSA_-OD_blank_). By comparing the value of OD_sample_-OD_blank_ to the standard curve, the amount of protein in each extract was calculated.

### Proteolytic digestion assay

The proteins were hydrolyzed using proteinase K and pepsin as described previously by Jeenkeawpieam *et al*. [22], Jeenkeawpieam *et al*. [24] and Sornwatana *et al*. [26] with slight modifications. The proteins were incubated at a 1:25 enzyme-to-protein ratio with porcine pepsin (P7000-100G, 250 units/mg) (Sigma-Aldrich, Missouri, USA) or proteinase K (Invitrogen, Thermo Fisher Scientific™, California, USA). We added 1 mg of porcine pepsin (250 units) to 25 mg of SYP1 protein in 10 mM CH_3_COONa buffer (pH 3.7). After determining the protein concentration of freeze-dried SYP1 dissolved in 1× PBS (pH 7.4), 25 mg of protein was hydrolyzed with 1 mg proteinase K (40 units/mg) to obtain SYP4. After 12 h of incubation at 37°C, the mixture was heat-inactivated for 10 min at 100°C. Both undigested and digested proteins were employed to test the antibacterial activity and the residual protein was detected using sodium dodecyl sulfate-polyacrylamide gel electrophoresis (SDS-PAGE).

### Sodium dodecyl sulfate-polyacrylamide gel electrophoresis

The profile of SYP and its hydrolysates were determined using SDS-PAGE. A 15% SDS-PAGE gel was loaded with 5 mg of each SYP for each run. The samples were electrophoresed in a mini-PROTEAN 2-D electrophoresis cell (Bio-Rad) using Tris–glycine buffer (pH 8.4) at a constant voltage (100 volts) for 2 h. The SDS-PAGE gels were stained with Coomassie Brilliant Blue (Thermo Scientific) to visualize the fingerprinting profile of the proteins [22–24].

### Screening the antibacterial activity of 500 μg SYP

We used the modified agar diffusion experiment to evaluate SYPs’ bacterial activity [24, 27]. The bacteria were cultured on Muller Hinton agar (MHA; Difco, USA) plates by covering the surface of these plates with 0.5 McFarland units of bacterial suspensions. After drilling 5 mm-wide wells into the agar plates using a sterile borer, the wells were filled with 100 μL of each extract containing 500 μg of each protein. The plates were incubated for 15–18 h at 35°C. The bacterial effects were reported as +, suppression with identifiable inhibition zones around the wells; −, no suppression with recorded inhibition zone diameters (mm). To evaluate the extracts’ antibacterial efficacy and compare it with the peptide/protein supernatants, we used a drug control or positive control (2.5 μg chloramphenicol). An inactivated enzyme suspension without any hydrolyzed protein was used as the negative control for SYPs. The following solutions were used as the negative controls for SYP1, SYP2, SYP3, and SYP4: 10 mM sodium acetate buffer (pH 5.2), 10 mM sodium acetate buffer (pH 3.7) with and without pepsin, and 1× PBS (pH 7.4) with and without proteinase K.

### Estimation of the minimum inhibitory concentration (MIC)/minimum bacterial concentration (MBC) of SYPs

According to the Clinical and Laboratory Standards Institute [24, 28], the extracts’ antibacterial activity was evaluated using the broth microdilution method against pathogens, such as *B. cereus*, *S. aureus*, *E. coli*, and *P. aeruginosa*. Three to five bacterial colonies were cultured in TSB (Difco), and incubated for 3–5 h at 35°C. A 96-well microtiter plate was filled with 50 μL of serially diluted extracts in the range of 1–512 μg/mL. Then, 50 μL of bacterial suspension containing 10^6^ colony-forming units/mL (CFU/mL) were transferred into a 96-well microtiter plate and incubated at 35°C for 15–18 h. The final concentration of the SYPs in each well was 0.5–256 μg/mL. Tetracycline was used as a positive control at a final concentration of 0.024–25 μg/mL per well. The growth control, located in column 11 of the 96-well plate, contained the growth medium with the bacterium, whereas growth medium alone was used as the negative control (column number 12).

Then, we evaluated the antibacterial activity using the visible qualitative approach by adding a blue-colored indicator dye resazurin (0.18% w/v, final concentration) (Sigma-Aldrich) to each well [29]. Color change of resazurin from blue to pink (resorufin) or colorless indicates bacterial growth (negative for antibacterial activity). Whereas, a blue or purple color indicates inhibition of bacterial growth (positive for antibacterial activity) [29]. Lack of inhibition at the maximum concentration tested (256 μg/mL) was recorded as the MIC at >256 μg/mL. The MIC values indicate the lowest concentration of the SYPs that can inhibit the growth of bacteria. All experiments were performed in triplicate.

To determine the MBC, the bacteria from all the wells that showed a blue or purple color were transferred to a TSA plate using the dropping plate method. The MBC was recorded as the lowest concentration of SYPs that showed no visible growth of bacteria colonies on the agar plates. An MBC value of >256 μg/mL was recorded if colonies were observed at the maximum concentration. All experiments were performed in triplicate [24].

### Inhibition of quorum sensing (QS) activity by SYPs against *Chromobacterium violaceum*

We determined the inhibitory effect of SYPs against the QS activity of *C. violaceum*, a biomonitoring strain, using agar well diffusion and microtube incubation assay as described previously. The QS ability is indicated by the production of the violacein pigment by the bacteria, resulting in violet-colored colonies [30].

First, we performed the visual qualitative assay using 256 μg of each protein hydrolysate against *C. violaceum* on an agar well diffusion plate. The MHA plates were spread with the overnight bacterial culture in TSB. Each SYP was added into an agar well at a final concentration of 256 μg/well and the plates were incubated at 35°C for 24 h. The QS inhibition zone was assessed by the formation of a colorless ring with viable cells around the well (non-pigmented zones), which indicated the anti-QS activity of the SYPs. The values were recorded in terms of the diameter of the QS inhibition zone and antibacterial activities (translucent zones). We used 10 mM CH_3_COONa (pH 5.2) as a negative control for SYP1, while those for SYP3, and SYP4 were 10 mM CH_3_COONa (pH 3.7) and 1× PBS (pH 7.4) with and without enzymes, respectively. The diameters of the anti-*C. violaceum* and QS inhibition zones were measured in mm and their mean and standard deviation (SD) were calculated. Each test was performed in three duplicates [30].

Next, we evaluated the SYP’s ability to inhibit the formation of violacein using a microtube incubation method. The bacteria were grown in a 1.5 mL microtube with TSB and a positive SYP that showed anti-QS activity against *C. violaceum* on an agar well diffusion plate at concentrations of 32, 64, and 128 μg/mL. The samples were placed in a shaking incubator (N-BIOTEK, Gyeonggi-do, Korea) at 35°C for 24 h with an agitation speed of 150 rpm. The growth of each culture was at an OD of 600 nm. After centrifuging the cell culture at 6,400× g for 5 min, the violacein precipitate was extracted from the bacterial cells by dissolving them in 100% dimethylsulfoxide (DMSO) and centrifuging them for another 5 min at 6,400× *g*. A 96-well microplate containing 100 μL of violacein supernatant was then added, and its absorbance was measured at 570 nm. Each test was performed in triplicate.

The following formula was used to determine the violacein production [30]:

%Violacein production = ([OD_570 nm_ SYPs treated cell-OD_570 nm_ negative control]/[OD_570 nm_ growth control-OD_570 nm_ negative control]) × 100.

### Effects of SYPs on biofilm formation of pathogenic bacteria

As the SYPs exhibited potent antibacterial efficacy, we evaluated their antibiofilm activity using the crystal violet test described by Tritripmongkol *et al*. [30] with slight modifications. Briefly, bacteria were grown in TSB enriched with 1% glucose, incubated at 37°C for 12–18 h, and diluted to 2 × 10^6^ CFU/mL. The 100 μL suspensions were added to a 96-well microtiter plate with 100 μL of each SYP. Then, the test plate was incubated at 37°C for 24 h. At OD 600 nm, the effects of SYPs on bacterial growth were assessed. After washing the wells twice with 1× PBS (pH 7.4) and drying, they were stained with 200 μL of a 0.1% crystal violet solution for 30 min. Then, they were air-dried after being rinsed twice with distilled water. The biofilms were dissolved using DMSO (200 μL). The inhibitory activity of the SYPs was assessed by measuring the biofilm growth at an OD of 570 nm using a microtiter plate reader (Thermo Scientific, Singapore). The formula used to determine the relative percentage of biofilm formation is ([mean OD_570 nm_ of treatment well-OD_570 nm_ of negative control]/[mean OD_570 nm_ of growth control well-OD_570 nm_ of nega­tive control]) × 100 [30]. The experiments were performed in triplicate.

### Effects of SYPs against *P. aeruginosa* pyocyanin

We qualitatively investigated the effects of the SYPs on *P. aeruginosa* pyocyanin. A *P. aeruginosa* overnight culture was subcultured into TSB + 1% glucose and adjusted to the 0.5 McFarland units. Aliquots of these bacterial solutions were added to a 96-well plate containing SYPs at 16–512 μg/mL and cultured for 24 h at 37°C. The growth medium was used as the negative control. Then, 10 μL of each suspension was dropped on *Pseudomonas* Isolation Agar (PIA) plates and incubated for 24 h at 37°C. All experiments were performed in triplicate. During the experiment, the same person inspected the PIA agar plates with an unaided eye. After each treatment, we recorded the colonies’ color on the PIA and compared them with that of the growth control. The SYP concentrations at which the colonies changed from blue–green to colorless in *P. aeruginosa* were considered effective against *P. aeruginosa* pyocyanin production.

### Effects of SYPs against staphyloxanthin in *S. aureus*

We qualitatively analyzed the SYPs’ effects on *S. aureus* staphyloxanthin [30]. We applied 0.5 McFarland standard number to an overnight *S. aureus* culture grown in TSB + 1% glucose. Then, aliquots of the bacterial suspension were added into tubes containing various concentrations of SYPs (16–512 μg/mL) and grown for 24 h at 37°C. Tryptic soy broth + 1% glucose was used as the negative control. Then, 10 μL suspension was then applied to the TSA plate and cultured for 24 h at 37°C. The experiments were performed in three duplicates. The same individual examined the TSA agar plates using an unaided eye. The golden–yellow colored *S. aureus* colonies were observed on the TSA plates and compared with the growth control. The SYP concentrations at which the *S. aureus* colonies changed from golden–yellow to colorless were considered effective against *S. aureus* staphyloxanthin production.

### Statistical analysis

All the statistical evaluations were done using statistical package for the social sciences (SPSS) version 16 (SPSS Inc., Chicago, USA). Model assumptions for normality and equal variances were checked using the Shapiro-Wilk test and Levene’s test, respectively. Results are expressed as mean ± SD for the analyses performed in triplicate. Significant differences were determined using a Tukey-honestly significant difference test, and p < 0.05 was considered statistically significant.

## Results

### Sodium dodecyl sulfate-polyacrylamide gel electrophoresis gel profiles of Sangyod rice seed protein and its hydrolysates

**Figure-1:**
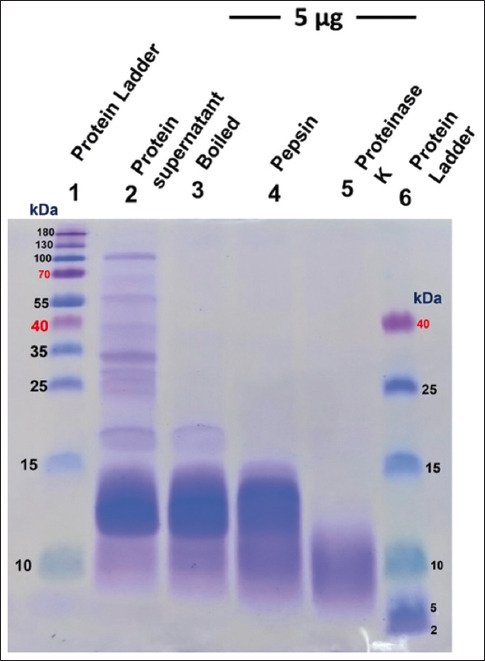
Detection of Sangyod rice seed protein patterns using sodium dodecyl sulfate-polyacrylamide gel electrophoresis (SDS-PAGE). 5 μg of each protein extract was separated in a 15% prepared SDS-PAGE gel and stained with Coomassie blue (Thermo Scientific™ Imperial™ Protein, Rockford). Standard protein marker from Thermo Scientific in Lane 1, Rockford, Illinois, USA. Sangyod rice seed protein extract (SYP1) is in Lane 2. Lane 3: Sangyod rice seed protein that has been heated, SYP1 is digested by Pepsin in Lane 4 and by Proteinase K in Lane 5. A low-range protein marker (Thermo Scientific, Rockford) is located in Lane 6.

Figure-1 shows the profiles of the Sangyod seed protein and its hydrolysate on a 15% SDS-PAGE gel. The bands between 10 and 140 kDa indicated SYP1. We observed three prominent bands at 10 kDa, 10–15 kDa, and 15–25 kDa for the heat-treated SYP2 proteins (by boiling SYP1 at 100°C for 10 min), indicating that most of the proteins had been destroyed. These three bands demonstrated their heat-resistant characteristics. The 10 kDa protein was predominant, as depicted in Figure-1. SYP3 and SYP4 are derived by treating SYP1 with pepsin and proteinase K, respectively. The pepsin-digested SYP3 displayed a distinct hydrolysate pattern; the size of the protein was visible at <15 kDa. The completely digested protein hydrolysate (<10 kDa) was observed in the SYP4 proteinase K-digested product (Figure-1).

### Screening the protein or peptides in SYP to investigate antibacterial efficacy

**Figure-2:**
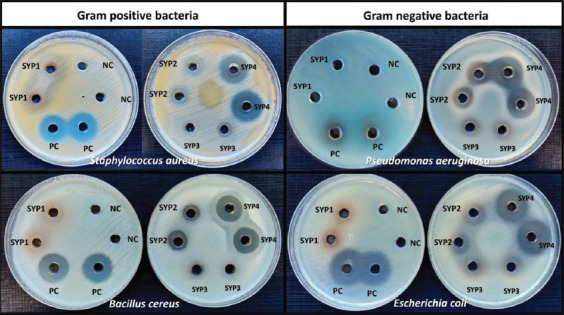
Screening of antibacterial activity of Sangyod protein hydrolysates at 500 μg protein with agar well diffusion. Protein extract (SYP1), heat-treated protein (SYP2), pepsin-hydrolyzed protein (SYP3), and proteinase K-hydrolyzed protein (SYP4) from Phatthalung Sangyod rice (*Oryza sativa* L.) seeds were tested for antibacterial properties. They were tested against four pathogenic bacteria using the agar well diffusion method. The Mueller Hinton agar’s surface was spread with bacterial cells, and the tested SYPs were employed to fill the wells. For 15–18 h, plates were incubated at 35°C. Positive (2.5 μg chloramphenicol) and negative (NC; 10 mM CH_3_COONa pH 5.2) controls are shown. Meanwhile, negative controls for SYP3 and SYP4 were 10 mM CH_3_COONa pH 3.7 and 1× phosphate-buffered saline pH 7.4 with and without enzyme, respectively. Negative controls did not affect bacterial growth.

The antibacterial activity of the SYP protein supernatant (SYP1) and their hydrolysates (SYP2–4) was tested against four medically important bacteria using the agar well diffusion method to determine whether their antibacterial activity was derived from proteins, peptides, or other biomolecules. Table-1 shows the inhibitory zones (in mm), indicating the antibacterial activity (Figure-2). We found three different types of inhibitory zones: “complete inhibition (+)” with no bacterial colonies (positive result); “partial inhibition” zone with a few surviving colonies that might be resistant to the test agent or might have regenerated after degradation of the test agent; and finally, no inhibition (−), which is a negative result.

**Table-1:** Antibacterial activity of Sangyod protein hydrolysates.

SYPs	Antibacterial activity (Mean ± SD of inhibition zone diameter (mm))			

	*Staphylococcus aureus*	*Bacillus cereus*	*Escherichia coli*	*Pseudomonas aeruginosa*
SYP1	-	-	-	-
SYP2	PI (12.25 ± 0.25)	+ (11.50 ± 0.58)	PI (10.25 ± 0.50)	+ (12.63 ± 0.25)
SYP3	PI (8.10 ± 0.25)	-	PI (9.63 ± 0.25)	PI (8.25 ± 0.25)
SYP4	+ (18.25 ± 0.50)	+ (16.88 ± 0.25)	+ (21.25 ± 0.50)	+ (18.75 ± 0.29)
Positive control: Chloramphenicol (2.5 μg)	+ (16.75 ± 0.50)	+ (18.13 ± 0.25)	+ (16.75 ± 0.25)	PI (8.15 ± 0.25)
Negative control:				
10 mM CH_3_COONa pH 5.2	-	-	-	-
10 mM CH_3_COONa pH 3.7				
10 mM CH_3_COONa pH 3.7+Pepsin	-	-	-	-
1×PBS pH 7.4	-	-	-	-
1×PBS pH 7.4+proteinase K	-	-	-	-

+=Complete Inhibition, -=No inhibition, PI=Partial inhibition, PBS=Phosphate-buffered saline, SD=Standard deviation

Notably, the crude protein (SYP1) did not inhibit the growth of any of the examined bacteria, but the heat-treated protein (SYP2) and the enzyme hydrolysates (SYP3 and SYP4) did. All of them, particularly SYP4, exhibited potent antibacterial activity against all tested pathogenic bacteria. SYP2 completely inhibited the growth of *B. cereus* and *P. aeruginosa*, while SYP2 and SYP3 partially suppressed *S. aureus* and *E. coli*. Surprisingly, SYP2, SYP3, and SYP4 (at 500 μg each) also reduced the staphyloxanthin (yellow-gold color) and pyocyanin (green color) pigmentation of *S. aureus* and *P. aeruginosa*, respectively, on the test plate (Figure-2).

### Minimum inhibitory concentration and MBC

**Figure-3:**
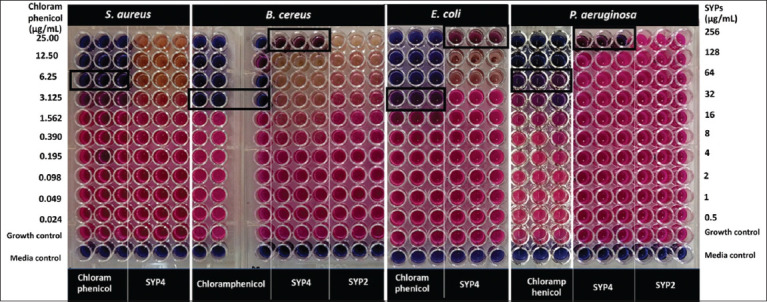
Minimum inhibitory concentration (MIC) values of Sangyod rice seed protein hydrolysates. 0.5 McF bacterial cells were treated with SYP2 and/or SYP4 in the final dilution range of 0.5–256 μg/mL and incubated for 15–18 h at 37°C. The pink and purple-dark blue colors in the MIC wells indicated no inhibition and inhibition of bacterial growth, respectively. The dark box indicated the MIC value.

Based on the results of the agar diffusion plate test, we used SYP2 and SYP4 at concentrations of 0.5–256 μg/mL to confirm the MIC and MBC values against *S. aureus*, *B. cereus*, *E. coli*, and *P. aeruginosa*. Table-2 and Figure-3 show the MIC and MBC values for all test pathogens bacteria. For *B. cereus*, *P. aeruginosa*, and *E. coli*, the MIC/MBC values of SYP4 were 256/>256 μg/mL, respectively. Nevertheless, SYP4 at a dose of 0.5–256 μg/mL did not inhibit *S. aureus*. We could not obtain the MIC or MBC values of SYP2 for *B. cereus* and *P. aeruginosa* within the test concentration range (Table-2 and Figure-3).

**Table-2:** Minimum inhibitory and MBC concentration values of Sangyod rice seed proteins on pathogenic bacteria (μg/mL).

Sangyod rice seed protein hydrolysates	Tested bacteria (MIC/MBC)			

	*Staphylococcus aureus*	*Bacillus cereus*	*Escherichia coili*	*Pseudomonas aeruginosa*
SYP2	NT/NT	> 256/NT	NT/NT	> 256/NT
SYP4	> 256/NT	256/> 256	256/> 256	256/> 256
Positive control: Chloramphenicol (0.024–25 μg/mL)	6.25/> 25	3.125/3.125	3.125/> 25	6.25/> 25

NT=Not tested, MIC=Minimum inhibitory concentration, MBC=Minimum bacterial concentration

### Antiquorum sensing activity of SYPs against *C. violaceum*

**Figure-4:**
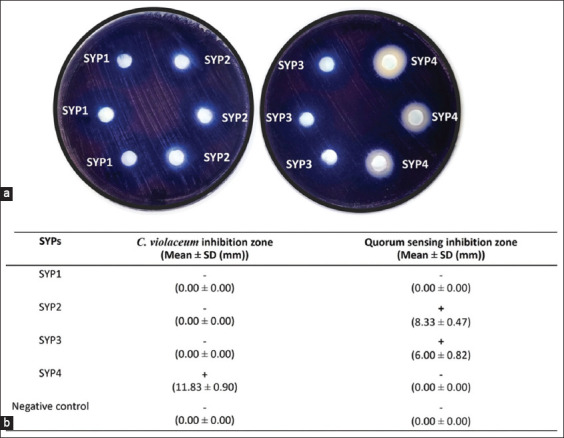
Visual qualitative assay by protein hydrolysates against *Chromobacterium violaceum* at 256 μg protein on an agar well diffusion plate. (a) Quorum sensing inhibition was assessed by the formation of a ring of colorless but viable cells around the well. The ring of purple colorless and viable organisms with a colorless bacterial colony (non-pigmented zones) indicated the anti-quorum sensing activity of the SYPs and antibacterial activities presented via translucent zones. Negative control is 10 mM CH_3_COONa pH 5.2 for SYP1. Meanwhile, negative controls for SYP3 and SYP4 were 10 mM CH_3_COONa pH 3.7 and 1× phosphate buffered saline pH 7.4 with and without enzyme, respectively. Negative controls did not affect bacterial growth. (b) The *C. violaceum* inhibition zone and quorum sensing inhibition zone diameters were measured in millimeters and given a mean ± Standard deviation.

Effective anti-QS activity of the extracts is reflected by the inhibition of violacein production without affecting bacterial growth. At a concentration of 256 μg of protein, SYP1 did not show anti-QS ability against *C. violaceum*. Nevertheless, SYP2 and SYP3 showed anti-QS ability at the same concentration without inhibiting the growth (Figure-4), with QS inhibition zones of 8.33 ± 0.47 mm and 6.00 ± 0.82 mm, respectively. Meanwhile, SYP4 did not show anti-QS at this concentration because it completely inhibited the growth of *C. violaceum*, with a huge inhibition zone of 11.83 ± 0.9 mm.

**Figure-5:**
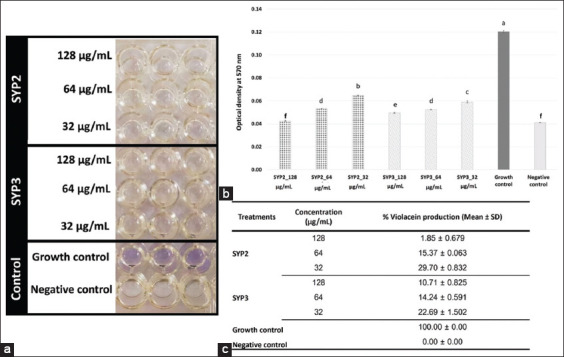
Inhibition of quorum sensing activity by protein hydrolysates against *Chromobacterium violaceum*. (a) 0.5 McF *C. violaceum* was aliquoted in a 1.5 mL test tube. They were treated with each SYP and incubated in a shaking incubator at 37°C for 24 h. Then the test tube was centrifuged. The cell was lysed with DMSO. After centrifugation, 100 μL of supernatant was transferred to a 96-well plate. The test plate was detected an optical density (OD)_570 nm_ value with the microreader plate. (b) Inhibition of the quorum sensing system by SYP2 and SYP3 showed a decrease in pigment production without growth. Means ± Standard deviation (SD) followed by different letters are significantly different by Tukey’s test (p < 0.05). (c) The determination of % Violacein production for each parameter was calculated by formula: ([OD_570_nm SYPs treated cell-OD_570_nm negative control]/[OD_570_nm growth control-OD_570_nm negative control]) 100×. All values are expressed as Mean ± SD.

Moreover, the inhibitory activity of the extracts showing anti-QS ability was further determined by quantifying the amount of violacein produced at OD_570__nm_ at 1/2, 1/4, and 1/8 concentrations of 256 μg/mL (Figure-5). The growth efficiency was checked to ensure that the *C. violaceum* growth in all treatment groups was similar to that of the control, indicating that the test concentration did not affect the growth. The production of violacein inside the *C. violaceum* cells indicates QS.

Our results showed a concentration-dependent reduction in violacein production (Figure-5). All concentrations (32, 64, and 128 μg/mL) of both SYP2 and SYP3 significantly reduced violacein production compared with untreated cells (growth control) (p < 0.05) (Figures-5a and 5b). Interestingly, at 128 μg/mL, SYP2 was most effective at inhibiting violacein synthesis in *C. violaceum*, which was not significant when compared to the negative control (p > 0.05) (Figure-5a and 5b). Moreover, only this treatment produced a tiny quantity of violacein at 1.85 ± 0.679% (Figure-5c).

### Antibiofilm formation activity of SYPs toward *S. aureus* and *P. aeruginosa*

**Figure-6:**
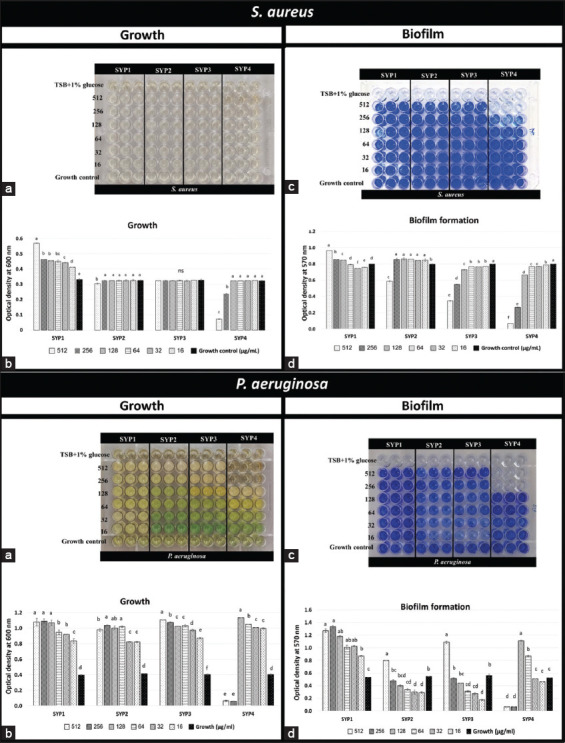
Effects of SYPs against bacteria-biofilm formation of *Staphylococcus aureus* (Upper) and *Pseudomonas aeruginosa* (Lower). (a and b) Effects of extracts from different parts of SYPs on growth and (c and d) the biofilm formation. (a) The pathogens were grown in tryptic soy broth plus 1% glucose. Then 0.5 McF bacterial cells were treated with each SYP for 24 h in a variety of concentrations and incubated at 37°C for 24 h. (b) Using a microplate reader, the test 96-well plates were evaluated for bacterial growth using an OD_600 nm_ score. Means ± Standard deviation (SD) followed by different letters are significantly different by Tukey’s test (p < 0.05). (c) Then the washed and dried test plates were stained with 0.1% crystal violet solution. DMSO was used to dissolve the Crytal violet-biofilm complex. (d) A microtiter plate reader (Thermo Scientific, Singapore City, Singapore) was used to measure the biofilm growth at an optical density (OD) of 570 nm. The biofilm formation was defined as the mean OD_570 nm_. Means ± SD followed by different letters are significantly different by Tukey’s test (p < 0.05).

We investigated the effect of SYPs (16–512 μg/mL) on the growth (Figures-6a and b) and biofilm formation (Figures-6c and d) of *S. aureus* and *P. aeruginosa*. The crude SYP1 protein promoted the growth of all test bacteria. The biofilm formation was also increased at a high concentration of SYP1, compared with growth control (p < 0.05). Then, we selected the concentrations that did not affect the growth of *S. aureus* to evaluate biofilm production, which were 16–256, 16–512, and 16–128 μg/mL for SYP2, SYP3, and SYP4, respectively.

SYP3 and SYP4 significantly reduced the biofilm formation of *S. aureus* at high concentrations (p < 0.05). Interestingly, all test concentrations of SYP3 did not inhibit *S. aureus* growth but significantly reduced its biofilm formation (p < 0.05). At 512 μg/mL, SYP3 showed the highest biofilm inhibition effect on *S. aureus* at 61.74%, followed by 256 (34.01%), 128 (9.53%), and 16–64 (3.92%–4.55%) μg/mL. Further, SYP4 had bactericidal and bacteriostatic ability against *S. aureus* at 256 and 512 μg/mL concentrations, respectively, so we excluded these concentrations from the antibiofilm activity test. However, at 16–128 μg/mL, biofilm production (1.42%–18.48%) was observed. While the biofilm production by *S. aureus* was significantly different (p < 0.05), it increased to 106.91%–108.91% when treated with 16–256 μg/mL of SYP2. Therefore, only SYP3 and SYP4 had antibiofilm activity against *S. aureus* (Figure-6).

Further, all SYPs significantly increased (p < 0.05) the biofilm formation of *P. aeruginosa* at high concentrations. Whereas MIC (256 μg/mL) and 2 × MIC (512 μg/mL) values of SYP4 completely inhibited *P. aeruginosa* growth. Confusingly, at concentrations below its MIC, SYP4 significantly increased the growth and biofilm formation (p < 0.05), which showed the same trend as other SYPs. Therefore, none of the SYPs inhibited *P. aeruginosa* biofilm formation.

### Effects of SYPs on pyocyanin production in *P. aeruginosa*

**Figure-7:**
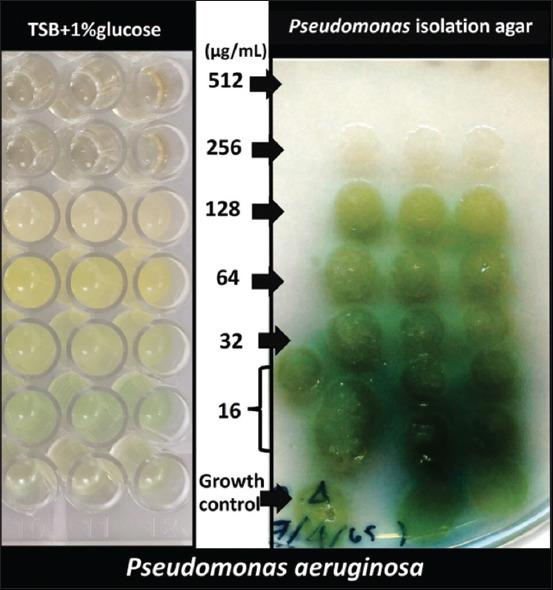
Effects of SYP4 on *Pseudomonas aeruginosa*’s synthesis of the pyocyanin pigment. *P. aeruginosa* was treated with SYP4 for 24 h in a variety of concentrations. A growth control with tryptic soy broth + 1% glucose was employed. After that, the bacterial suspension was dropped onto *Pseudomonas* isolation agar (PIA). A visual qualitative assay for inhibition of *P. aeruginosa* pyocyanin production was detected. The color change of the colonies was observed and compared with the growth control.

As shown in Figure-7, we visually assessed the efficacy of different concentrations of SYP4 on the inhibition of the production of a blue–green pigment, pyocyanin by *P. aeruginosa* ATCC27853. At 256 μg/mL of SYP4, colorless *P. aeruginosa* colonies were observed, indicating that SYP4 completely inhibited pyocyanin production compared with growth control. Moreover, other SYPs, at 16–512 μg/mL, did not inhibit pyocyanin production by *P. aeruginosa*.

### Effects of SYPs on staphyloxanthin in *S. aureus*

**Figure-8:**
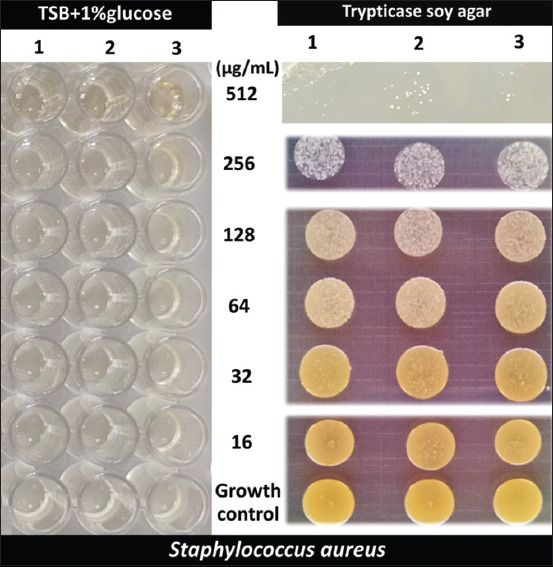
Visual qualitative assay for inhibition of staphyloxanthin in *Staphylococcus aureus* by SYP4. *S. aureus* was treated for 24 h with SYP4 at varying doses. The growth control utilized was tryptic soy broth + 1% glucose. After that, the bacterial suspension was dropped onto tryptic soy agar. The visual qualitative colonies’ colors changes were assessed and compared with the growth control.

Staphyloxanthin, a yellow pigment produced by *S. aureus*, is also a virulence factor. We investigated the effect of all SYPs on staphyloxanthin production in *S. aureus* by incubating the bacterium in a medium supplemented with the SYPs, followed by culturing on the agar plate. The results demonstrated that only SYP4 inhibited staphyloxanthin production. At 256 μg/mL, SYP4 completely inhibited staphyloxanthin production in *S. aureus* compared to the growth control (Figure-8).

## Discussion

Rice is a staple diet for over half of the world’s population, who are mostly Asian. In Thailand, various varieties of rice are grown. In our earlier research, we showed that the hydrolysates from the Phatthalung Sangyod rice seed protein, obtained by boiling, pepsin, and proteinase K digestion, have antifungal action against several opportunistic fungi, including *Cryptococcus neoformans*, which affects both humans and animals. In addition, studies have shown that the proteinase K-hydrolyzed protein is non-hemolytic toward animal erythrocytes [22]. Here, we investigated the antibacterial and antivirulence factors activities of the protein hydrolysates from Phatthalung Sangyod rice seed against *B. cereus*, *S. aureus*, *E. coli*, and *P. aeruginosa*, which cause foodborne infections. In other words, we investigated whether rice can be used as an antibiotic alternative to combat the incidence of antibiotic resistance in the future.

To determine whether the antibacterial activity in the SYP was derived from proteins, peptides, or other biomolecules, we assessed the antibacterial efficacy of SYP protein supernatant (SYP1) and its hydrolysates (SYP2–4) against four medically significant bacteria using the agar well diffusion method. Our findings revealed that while SYP1 did not affect bacteria growth, its hydrolysates, SYP2, SYP3, and SYP4 exhibited antibacterial activity. As neither the protein extract nor the residual phenolic compounds from the Sangyod rice possessed any antibacterial properties, we concluded that the main antibacterial components were the peptides or protein hydrolysates. Bioactive peptides are produced by several processes, including (1) heating; (2) acid–base hydrolysis; (3) enzymatic hydrolysis; and (4) microbial activity (mostly in fermented foods) [31, 32]. Proteins that have been hydrolyzed into complex peptide mixtures are more easily digestible and bioavailable than whole proteins [33]. During gastrointestinal digestion, fermentation, and food processing, these bioactive peptides, which are encoded within the native protein sequences, are produced [34]. Thus, Sangyod rice seed protein hydrolysates obtained by heating or treatment with enzymes, such as proteinase K (under alkaline conditions), similar to the human or animal intestine, and pepsin enzyme (under acidic conditions), similar to the stomach, can produce bioactive peptides with antibacterial activity against both Gram-negative and -positive bacteria [21, 31–34].

So far, only one study by Ditsawanon *et al*. [21] has explored the efficacy of these antibacterial protein hydrolysates and showed that 100 μg/mL of pepsin-hydrolyzed Sangyod rice seed protein inhibited the growth of *S. aureus*, *P. aeruginosa*, and *E. coli*. However, we showed that 500 mg of SYP3 could only partially inhibit these bacteria, which might be due to variations in pH and concentration of the sodium acetate solution used for protein extraction. In addition, as the samples were heated at 121°C before the pepsin hydrolysis, a highly concentrated and strongly acidic extraction buffer was required to extract the bioactive peptides using pepsin hydrolysis with good antibacterial activity. Furthermore, the residual heat-tolerant proteins were also more likely to break down into bioactive peptides at 121°C than the native crude protein. Therefore, to derive the bioactive peptides from the Sangyod rice seed, they need to be cooked and digested in the gastrointestinal tract of animals and humans [22].

Among all the SYP protein hydrolysates, the proteinase K-treated one (SYP4) showed the strongest antibacterial activity. At 500 μg, SYP4 strongly inhibited all test bacteria. Moreover, the MIC/MBC values of SYP4 against *B. cereus*, *P. aeruginosa*, and *E. coli* were 256/>256 μg/mL, though we could not obtain a MIC value against *S. aureus* at concentrations ranging from 0.5 to 256 μg/mL. SYP4 exhibited potent bactericidal (>256 μg/mL) and bacteriostatic (at 256 μg/mL) activity against tested pathogenic bacteria. In addition, SYP2 and SYP3 showed negligible bactericidal activity. At 500 μg protein, SYP2 inhibited the growth of *P. aeruginosa*, and *B. cereus*, and partially inhibited that of *S. aureus* and *E. coli*. However, the MIC/MBC values could not be obtained for SYP2 at concentrations between 0.5 μg/mL and 256 μg/mL. Further, all test strains were partially suppressed by SYP3, probably because pepsin cannot effectively digest low-molecular-weight protein fragments to produce hydrolysates with effective antibacterial properties. Unlike low-molecular-weight proteins or short peptides from protein hydrolysate, which are digested by proteinase K, they have been shown to possess stronger bioactivity than high-molecular-weight proteins [32]. As shown in Figure-1, the SDS-PAGE gel profile for SYP4 consisted of peptides below 10 kDa, which showed the maximum antibacterial activity, followed by SYP2 and SYP3. Muangrod *et al*. [35] explained that the raw material, hydrolysis process, the type and activity of the enzyme, and extraction time influenced protein size. Our results confirmed that the proteins hydrolyzed using different enzymes have different bioactivities, such as antibacterial or antifungal activities [22]. Therefore, increased hydrolysis time, increased enzyme concentration, and protein hydrolysate purification may be required to improve the bioactivity of the Sangyod rice seed protein hydrolysate. However, as the native or raw crude protein (SYP1) does not possess efficient antibacterial properties, it stimulates bacterial growth depending on its concentration. Therefore, in Thailand, raw Sangyod rice seeds can be used as an affordable low-cost carbon source for producing bacterial mediums. Due to their high starch content, raw rice seeds are good sources of carbon to support the growth of microorganisms to ferment several products [36, 37].

We did not characterize the mode of action (MOA) by which SYPs affect bacterial cells. We observed that Gram-positive and -negative bacteria were completely inhibited by SYP4. Based on this, the MOA might be unrelated to the peptidoglycan and outer membrane components of the bacterial cell wall, which differentiate the Gram-positive from Gram-negative bacteria. Several studies have shown that antimicrobial peptides (AMPs) are mostly positively charged amphipathic molecules that can target and kill bacteria utilizing two different MOAs: (1) membrane rupture followed by cell lysis and death, and (2) entry into cells without rupturing the cell membrane and inhibiting crucial intracellular processes by binding to intracellular proteins or nucleic acids [38]. The second MOA is consistent with the properties of SYP4 that were hydrolyzed with proteinase K under slightly alkaline conditions (pH 7.4). Most bioactive AMPs from SYP4 are most likely positively charged. As transmembrane pore formation is not the only method for killing microbes. It is possible that SYP4 kills the microbes using other strategies, including inhibiting cell wall and/or nucleic acid synthesis, activating the autolytic enzyme system, or acting synergistically with another host’s innate immune molecules [39].

Numerous antibiotics have been used to treat bacterial infections throughout several decades. However, according to Uddin *et al*. [40], the rate of discovering novel antibiotics has considerably declined. In addition, the rise of multidrug-resistant bacteria has limited the effective use of bactericidal agents [40, 41]. To prevent the potential emergence of drug resistance, antivirulence approaches that reduce the production of virulence factors without harming bacterial growth have gained significant research interest [42, 43]. In this study, we examined the inhibitory effects of SYPs on several virulence mechanisms, such as QS, biofilm formation, and pigment production. National Institutes of Health reported that biofilm formation is associated with 60%–80% of all microbial infections [44]. For biofilm formation, some bacteria use QS systems to synchronize their gene expression to create a biofilm [45]. Bacterial quorum sensing (QS) relies on the generation, secretion, and detection of autoinducer (AI) signals to control gene expression in response to variations in population density [45]. For instance, QS in Gram-negative bacteria is mediated by acyl-homoserine lactones (AHLs) [46], which are found in various forms depending on the length and functionalization of the acyl side chain. Although its function in cell signaling is still debatable, small peptides control QS-mediated gene expression in Gram-positive bacteria and AI-2 mediates QS in both Gram-positive and Gram-negative bacteria [47]. Quorum sensing regulates several physiological processes, including bioluminescence, the release of virulence factors, biofilm formation, pigment production, and antibiotic resistance. Quorum sensing has been shown to control biofilm development in numerous bacteria [30, 45]. Both eDNA release and biofilm structure are controlled by AHL QS systems in the *P. aeruginosa* PAO1 strain [48]. In *S. aureus*, the gene agrD encodes for AgrC, which is a peptide involved in QS. AgrC, in turn, detects and activates the regulator AgrA [49], which controls the transcription of genes encoding proteases required for biofilm dispersal, as shown by Bole and Horswill [49].

Here, we showed that the SYPs, mainly SYP2 and SYP3, interfere with QS activity in *C. violaceum* and perform quorum-quenching (QQ) during biofilm formation in *S. aureus*. Both SYP2 and SYP3 (at a minimum of 32 μg/mL) exhibited anti-QS activity in *C. violaceum*. Interestingly, 128 μg/mL of SYP2 significantly inhibited violacein production. While, at 16–512 μg/mL, SYP3 also demonstrated potent antivirulence mechanisms against biofilm production in *S. aureus*. Moreover, at 16–128 μg/mL, SYP4 also demonstrated biofilm inhibition of *S. aureus*, although this effect was unrelated to QS inhibition of *C. violaceum*. This might be because SYP might contain a bioactive phytochemical that inhibits disease-causing genes by interfering with virulence proteins linked to QS and biofilm formation [50]. According to Degrassi *et al*. [51], *O. sativa* (rice) plants release AHL-mimic compounds that can activate several QS biosensors. In addition, according to Moradi and Hadi [52], the QQ activity of some plants disrupts the bacterial QS signals in various ways, including (1) preventing the synthesis of signaling molecules, (2) inactivating or enzymatically destroying signaling molecules or preventing their accumulation to a threshold value, (3) interfering or competing with the binding of signal receptors and/or their analogs, and (4) blocking target genes that need to be activated. For instance, the RNAIII inhibiting peptide, a QS inhibitor, suppressed the TRAP/agr system and biofilm formation in *S. aureus* [53]. Additionally, proteins and/or specific chemicals, including quercetin, catechin, rosmarinic acid, limonoid, ichangin, apigenin, kaempferol, and naringenin have been demonstrated to affect biofilm-associated infections [54]. Therefore, the protein hydrolysates from Sangyod rice seeds might act as natural QS inhibitors or QQ quenchers.

Furthermore, SYPs could suppress pyocyanin and staphyloxanthin production in *P. aeruginosa* and *S. aureus*, respectively. These pigments act as virulence factors in *P. aeruginosa* and *S. aureus* and can be potentially novel targets for antivirulence therapy [55]. The colors produced by *P. aeruginosa* have been shown to be influenced by its virulence and tolerance to cationic antibiotics [56]. However, although *P. aeruginosa* produces four types of pigments, pyocyanin, pyoverdine, pyorubrin, and pheomelanin, only pyocyanin, a soluble blue–green pigment, has been investigated [56]. According to Behzadi *et al*. [56], this pigment has been identified as a crucial component in *Pseudomonas* pathogenicity, particularly in the skin, soft tissue, and lung invasive infections. While staphyloxanthin, a yellow to gold–orange pigment, is recognized as a crucial virulence component for *S. aureus* [55]. The current findings demonstrated that, at 256 μg/mL, only SYP4 inhibited the synthesis of pyocyanin and staphyloxanthin in *P. aeruginosa* and *S. aureus*, respectively. Although at 0.5– 512 μg/mL, SYP2 and SYP3 could not suppress pigment production in both these bacteria, they could do so at high concentrations (500 μg protein or 5,000 μg/mL concentration), as shown on the agar well diffusion test. These findings suggest that SYP4 could regulate or hinder the synthesis of pyocyanin and staphyloxanthin in both bacteria. Based on this, we speculated that *S. aureus* and *P. aeruginosa* might be unable to colonize and persist in the host after being treated with Sangyod rice. In addition, we previously showed that SYP4 showed antifungal efficacy and was non-cytotoxic to canine red blood cells [22]. Therefore, cooked Sangyod rice can be safely used for consumption by humans or animals with gastrointestinal diseases to prevent bacterial infection and suppress the virulence factors from gastrointestinal pathogens [57, 58].

To gain a deeper understanding of their potential as antimicrobial agents, research efforts should focus on the isolation and characterization of pure bioactive peptides from the Sangyod rice seed protein hydrolysates. Conducting advanced *in vitro* and *in vivo* analyses can help understand the mechanism, MOA, and biosafety of the identified peptide fractions against microbial strains.

## Conclusion

The Phatthalung Sangyod protein hydrolysates that were heated (SYP2), and hydrolyzed using pepsin (SYP3), and proteinase K (SYP4) significantly reduced the growth of pathogenic bacteria that cause foodborne infections and also inhibited their virulence factors, such as QS in *C. violaceum*, biofilm formation in *S. aureus*, and/or pigment production in *S. aureus* and *P. aeruginosa*. Our experiments showed that SYP4 outperformed all protein hydrolysates in terms of bacteriostatic and bactericidal efficacy and antipigment production. The strongest QS inhibition was observed in SYP2, while SYP3’s primary function is to prevent antibiofilm development. Based on these findings, we conclude that they can be used as natural alternatives for antimicrobials or functional foods during food processing to control the infection caused by bacterial pathogens in humans and animals.

## Authors’ Contributions

JJ and PR: Conceptualization and writing-review and editing. JJ, PR, SS, WM, MP, PS, IT, JL, and KP: Methodology and investigation. JJ, PR, and MP: Writing-original draft preparation. JJ: Funding acquisition. All authors have read, reviewed, and approved the final manuscript.
